# Exploring the socio-economic determinants of educational inequalities in diarrhoea among under-five children in low- and middle-income countries: a Fairlie decomposition analysis

**DOI:** 10.1186/s13690-021-00639-8

**Published:** 2021-06-24

**Authors:** Adeniyi Francis Fagbamigbe, Olukemi Grace Adebola, Natisha Dukhi, Omon Stellamaris Fagbamigbe, Olalekan A. Uthman

**Affiliations:** 1 Department of Epidemiology and Medical Statistics, Faculty of Public Health, College of Medicine, University of Ibadan, Ibadan, Nigeria; 2 Warwick Centre for Global Health, Division of Health Sciences, University of Warwick, Coventry, UK; 3 General Studies Unit, School of Sciences, Federal University of Technology, Akure, Nigeria; 4 Human and Social Capabilities Division, Human Sciences Research Council, Cape Town, South Africa; 5 Techmodia, West Sussex, UK; 6 Portsmouth Business School, Faculty of Business and Law, University of Portsmouth, Portsmouth, UK; 7 Division of Epidemiology and Biostatistics, Department of Global Health, Faculty of Health Sciences, Stellenbosch University, Francie van Zijl Drive, Tygerberg, 7505 Cape Town, South Africa

**Keywords:** Diarrhoea, Educational inequalities, Fairlie decomposition, Low and middle-income countries, Risk difference

## Abstract

**Background:**

What explains the underlying causes of educational inequalities in diarrhoea among under-five children in low- and middle-income countries (LMIC) is poorly exploited, operationalized, studied and understood. This paper aims to assess the magnitude of educational-related inequalities in the development of diarrhoea and decompose risk factors that contribute to these inequalities among under-five children (U5C) in LMIC.

**Methods:**

Secondary data of 796,150 U5C from 63,378 neighbourhoods in 57 LMIC was pooled from the Demographic and Health Surveys (DHS) conducted between 2010 and 2019. The main determinate variable in this decomposition study was mothers’ literacy levels. Descriptive and inferential statistics comprising of bivariable analysis and binary logistic multivariable Fairlie decomposition techniques were employed at *p* = 0.05.

**Results:**

Of the 57 countries, we found a statistically significant pro-illiterate odds ratio in 6 countries, 14 showed pro-literate inequality while the remaining 37 countries had no statistically significant educational-related inequality. The countries with pro-illiterate inequalities are Burundi (OR = 1.11; 95% CI: 1.01–1.21), Cameroon (OR = 1.84; 95% CI: 1.66–2.05), Egypt (OR = 1.26; 95% CI: 1.12–1.43), Ghana (OR = 1.24; 95% CI: 1.06–1.47), Nigeria (OR = 1.80; 95% CI: 1.68–1.93), and Togo (OR = 1.21; 95% CI: 1.06–1.38). Although there are variations in factors that contribute to pro-illiterate inequality across the 6 countries, the overall largest contributors to the inequality are household wealth status, maternal age, neighbourhood SES, birth order, toilet type, birth interval and place of residence. The widest pro-illiterate risk difference (RD) was in Cameroon (118.44/1000) while the pro-literate risk difference was widest in Albania (− 61.90/1000).

**Conclusions:**

The study identified educational inequalities in the prevalence of diarrhoea in children with wide variations in magnitude and contributions of the risk factors to pro-illiterate inequalities. This suggests that diarrhoea prevention strategies is a must in the pro-illiterate inequality countries and should be extended to educated mothers as well, especially in the pro-educated countries. There is a need for further studies to examine the contributions of structural and compositional factors associated with pro-educated inequalities in the prevalence of diarrhoea among U5C in LMIC.

## Background

Diarrhoea, the passage of loose or liquid stools three or more times per day, remains a major health challenge especially among under-five children (U5C) [1] in the low- and middle-income countries (LMIC). In low-income countries, children under five years, experience on average three episodes of diarrhoea every year and each episode deprive the child of the nutrition required for growth [2]. In 2016, UNICEF reported that about a quarter of the nearly 6 million children who do not live beyond the age of 5 dies from diarrhoea or pneumonia [3]. The UNICEF maintains that LMIC is home to 62% of the world’s U5C population but account for more than 90% of the global diarrhoea death [4]. It has been delineated that the fate of U5C is determined by inequities especially with regards to the regions of the world in which they are born and diarrhoea is prevalent and concentrated within the poorest of the poor populations of the world [4]. The report also indicated that 70 million children die before reaching their fifth birthday and children in LMIC are ten times more likely to be affected than children in high-income countries. UNICEF and WHO declared diarrhoea as the second leading killer of U5C [1]. Acute diarrhoea diseases remain a leading cause of global morbidity and mortality particularly among young children in resource-limited countries with malnourished children at higher odds [2, 5]. Children continue to face widespread regional disparities in their chances of survival and sub-Saharan Africa remains the region with the highest under-five mortality rate in the world [6]. This is an alarming reminder of the exceptional vulnerabilities of children in developing countries.

There are environmental, social and economic risk factors of diarrhoea in LMIC as revealed from past studies. Prominent among these factors are illiteracy, gross inadequacy of safe drinking water, sanitation facilities, and good hygiene [1, 2, 7–9]. Other studies revealed that in sub-Saharan Africa, socioeconomic factors such as place of residence, mother’s educational level, household wealth strongly impacted U5C mortality [3, 10, 11]. As opined by Budhathoki et al., cultural and societal values and income level are determinants for diarrhoea in U5C particularly in developing countries [12]. Among the social dimensions responsible for diarrhoea-related child mortality, maternal education has consistently remained significant, with children of less-educated mothers being considerably at higher risk [8, 9]. Also, reiterated is the fact that maternal education, lack of availability of latrine, lack of maternal handwashing and rural area residence correlated with childhood diarrhoea significantly [13].

Replete in literature is the importance of mother’s level of education in the development of diarrhoea in U5C. For instance, Workie et al. asserted that as a primary caregiver all over the world, mothers play a central role in the management and prevention of diarrhoea [14]. Bohra et al. affirmed that maternal education plays an important role in determining access to water and sanitation, and inequalities in child mortality arising due to differential access especially in low-and middle-income countries [15]. Woldu et al. averred that mother’s education and household economic status were significantly associated with high mortality in U5C due to diarrhoea diseases [16], Budhathoki et al. reaffirmed that children whose mothers had secondary education or higher have lower odds of diarrhoea in comparison to children of mothers with lower educational attainment [12]. A recent study showed that children whose mothers have never attended primary school, who lives where faeces are exposed and in buildings with earthen floors were significantly associated with diarrhoea [17]. UNICEF had linked women’s education level to a child’s health and survival among the U5C [18]. Literature is satiated with factors that increase the susceptibility of U5C to diarrhoea especially in LMIC, with mother’s educational level most emphasized. Nonetheless, what explains the underlying causes of educational inequalities in diarrhoea among under-five children in low- and middle-income countries (LMIC) is poorly exploited, operationalized, studied and understood.

Inequalities in maternal education are a critical condition for the development of diarrhoea in U5C which remains a persistent burden among the LMIC of the world. However, the factors responsible for such disparities such as having diarrhoea have not been well studied. There is a need to understand such factors to proffer workable solutions. An understanding of the factors responsible for maternal educational inequalities is a necessity if there will be any remarkable progress to forestall the burden of diarrhoea among U5C. Such an understanding would inform interventions for diarrhoea prevention. We aimed to conduct multi-country analyses with efforts to decompose the educational-related inequalities in the development of diarrhoea among U5C in LMIC and also identified the risk factors contributing to these inequalities. Strategies to prevent diarrhoea are recommended, many of which emphasize the need to explore individual-, household and neighbourhood-level and country-specific factors.

## Methods

### Study design and data

We pooled the Demographic and Health Surveys (DHS) data from 57 LMIC in this study using the most recent successive DHS conducted within the last ten years (2010–2019) which captured information on diarrhoea experience among U5C and available as of April 2020. The DHSs are cross-sectional and nationally representative population-based household survey conducted periodically across the LMIC. The DHS uses a multi-stage stratified sampling design based on the states/divisions/regions, district and clusters peculiar to each country. In each of the countries, the households are the sampling units and are selected from the clusters which are the primary sampling units (PSU) [19, 20]. The DHS computes sampling weights to account for unequal selection probabilities within each cluster as a result of unequal sample sizes of the clusters. The application of the sampling weights ensured that the survey findings fully represent the target populations. A similar set of protocols, standardized questionnaires, similar interviewer training, supervision, and implementation were used in all the countries. The full details of the sampling methodologies are available at dhsprogram.com. Amongst others, the DHS collects data on children health care including common diseases, treatments, and care for all U5C of the sampled women. In all, we extracted the data of 796,150 U5C from 63,378 neighbourhoods in 57 LMIC across the globe.

### Dependent variable

The outcome variable in this study is the recent experience of diarrhoea. Diarrhoea is defined as “passage of liquid stools three or more times a day” [21, 22] and “recent experience of diarrhoea” as having any of the symptoms of diarrhoea within two weeks before the date of the interview [23]. The mothers were asked if any of their U5C had diarrhoea within two weeks preceding the survey. The responses were binary: Yes or No.

### Main determinant variable

The main determinate variable in this decomposition study is mothers’ literacy levels: illiterate or literate. We used mothers’ reported education as a proxy for literacy in this study. Literacy, a key skill and an important measure of a population’s level of education, is the ability to both read and write a short, simple statement about one’s own life [24]. We, therefore, categorized education as having no formal education (Illiterate) and educated (can read and write: have a minimum of completed primary education - Literate).

### Independent variables

The independent variables consist of individual-level and neighbourhood-level factors.

### Individual-level factors

The individual-level factors comprises children, mothers’ and the households’ characteristics. Childs’ characteristics: sex (male versus female), age in years (< 12 months and 12–59 months), weight at birth (average+, small and very small), birth interval (firstborn, < 36 months and > =36 months) and birth order (1, 2, 3 and 4+). Mothers’ characteristics: maternal age (15–24, 25–34, 35–49), marital status (never, currently and formerly married), employment status (working or not working). Households’ characteristics: access to media (at least one of radio, television or newspaper), sources of drinking water (improved or unimproved), toilet type (improved or unimproved), cooking fuel (clean fuel or biomass), housing materials (improved or unimproved).

### Neighbourhood-level factors

The clusters are the PSUs in the DHS sampling technique. Typically, people of the same cluster share similar contextual factors [19, 20]. We used the word “neighbourhood” to describe the clustering of the children within the same geographical cluster and “neighbours” as the members of the same cluster. The PSUs were identified using the most recent census in the respective countries. In this study, we considered living in rural areas and neighbourhood socioeconomic status (SES) as community-level variables. We computed the neighbourhood SES using the principal component analysis method, comprising of the proportion of respondents within the same neighbourhood who are from poor households and are not currently employed.

### Statistical analyses

**Fig. 1:**
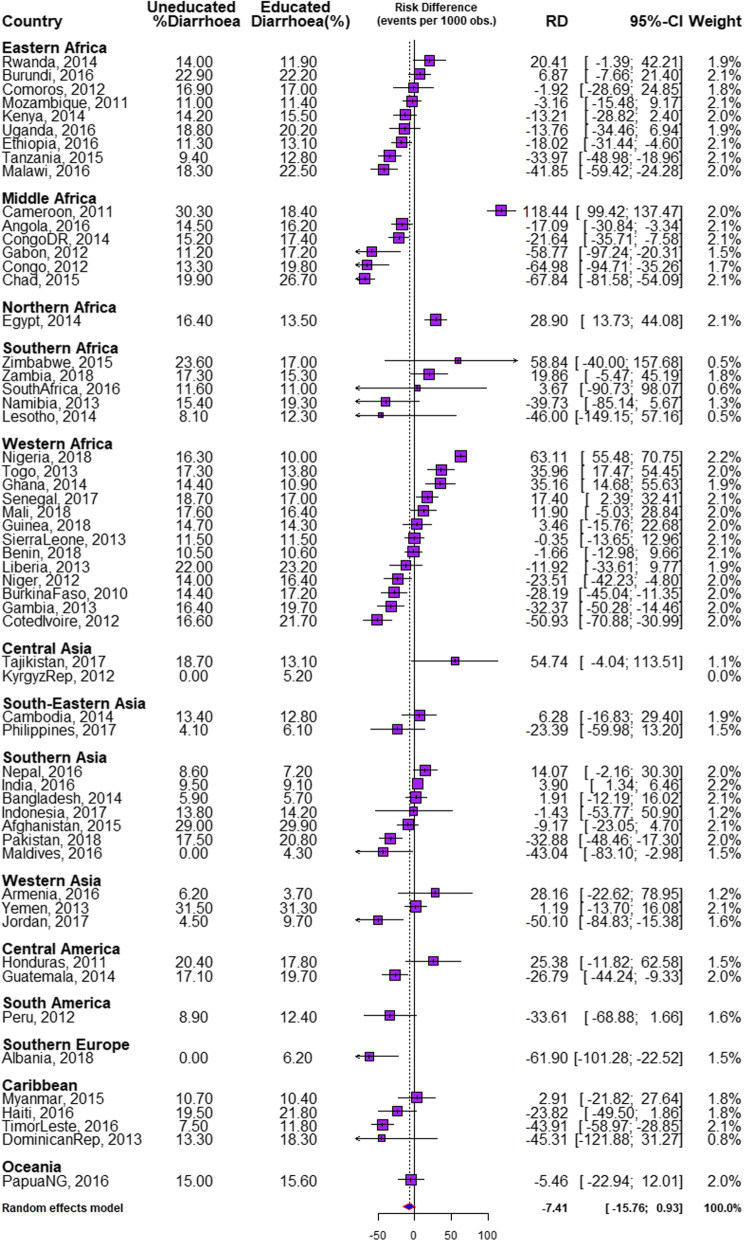
Risk difference in the prevalence of diarrhoea between children from illiterate and literate mothers by countries

**Fig. 2:**
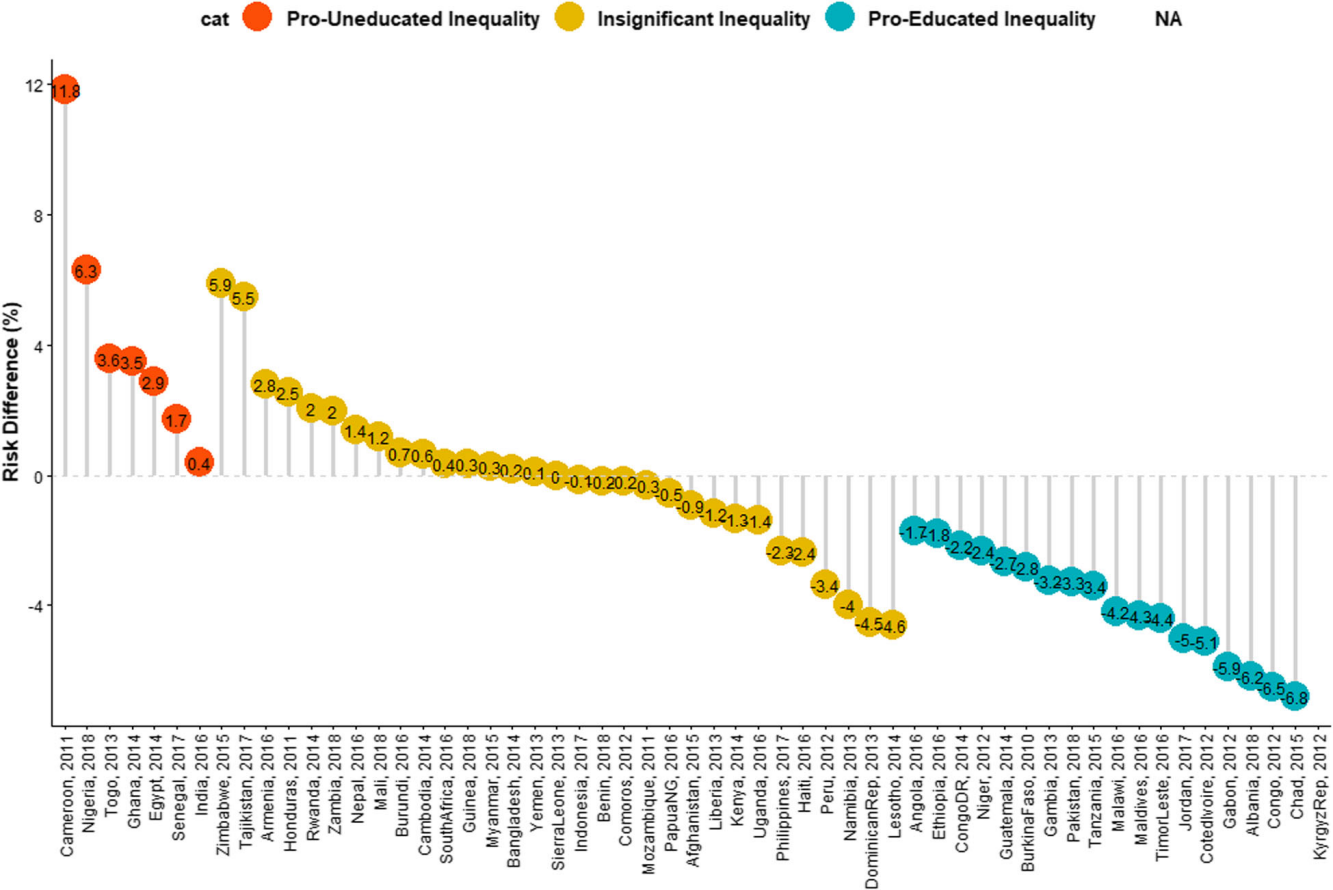
Risk difference between children born to illiterate and literate mothers in the prevalence of diarrhoea by countries

**Fig. 3:**
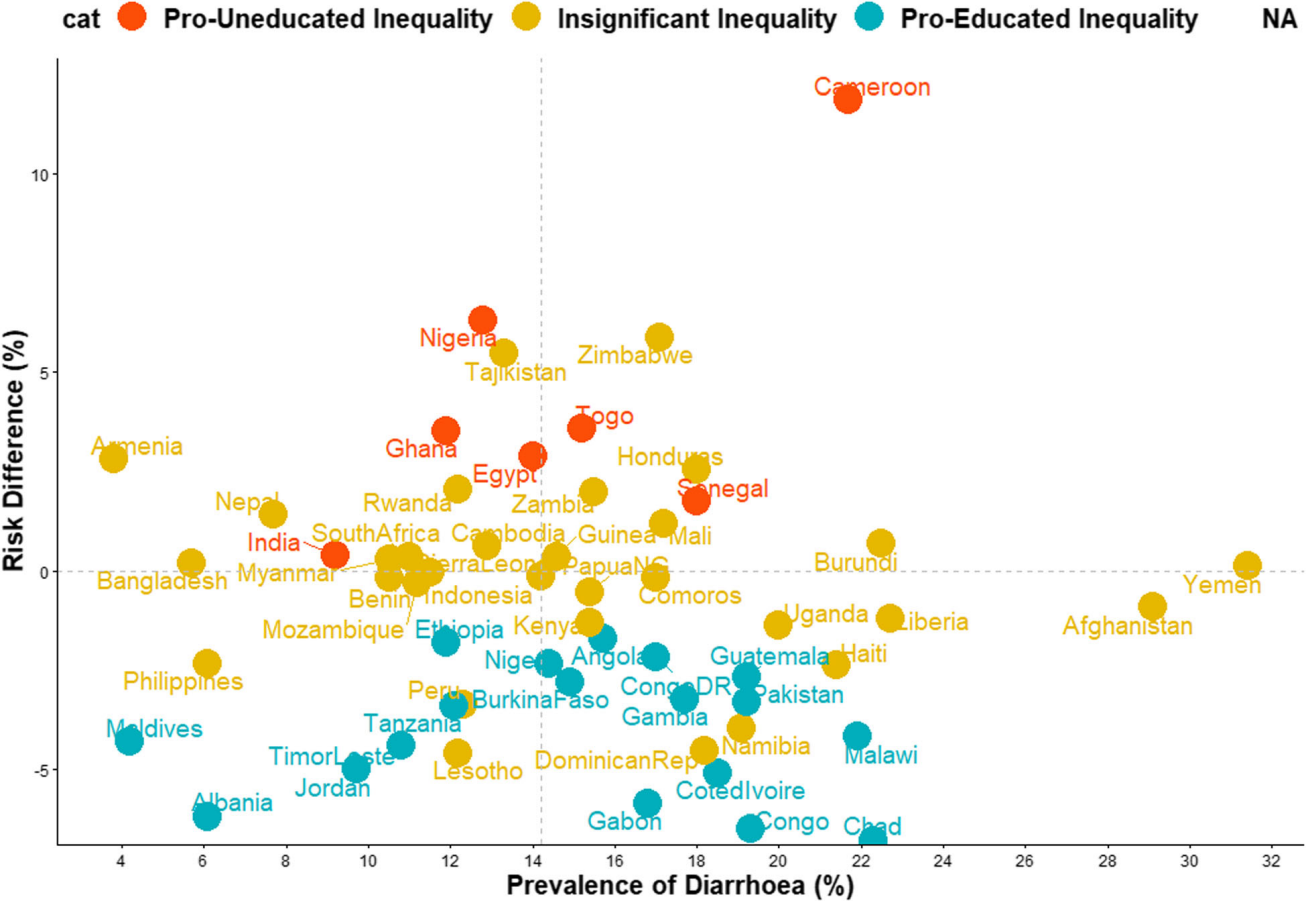
Scatter plot of the rate of diarrhoea and risk difference between children born to illiterate and literate mothers in LMIC

**Fig. 4:**
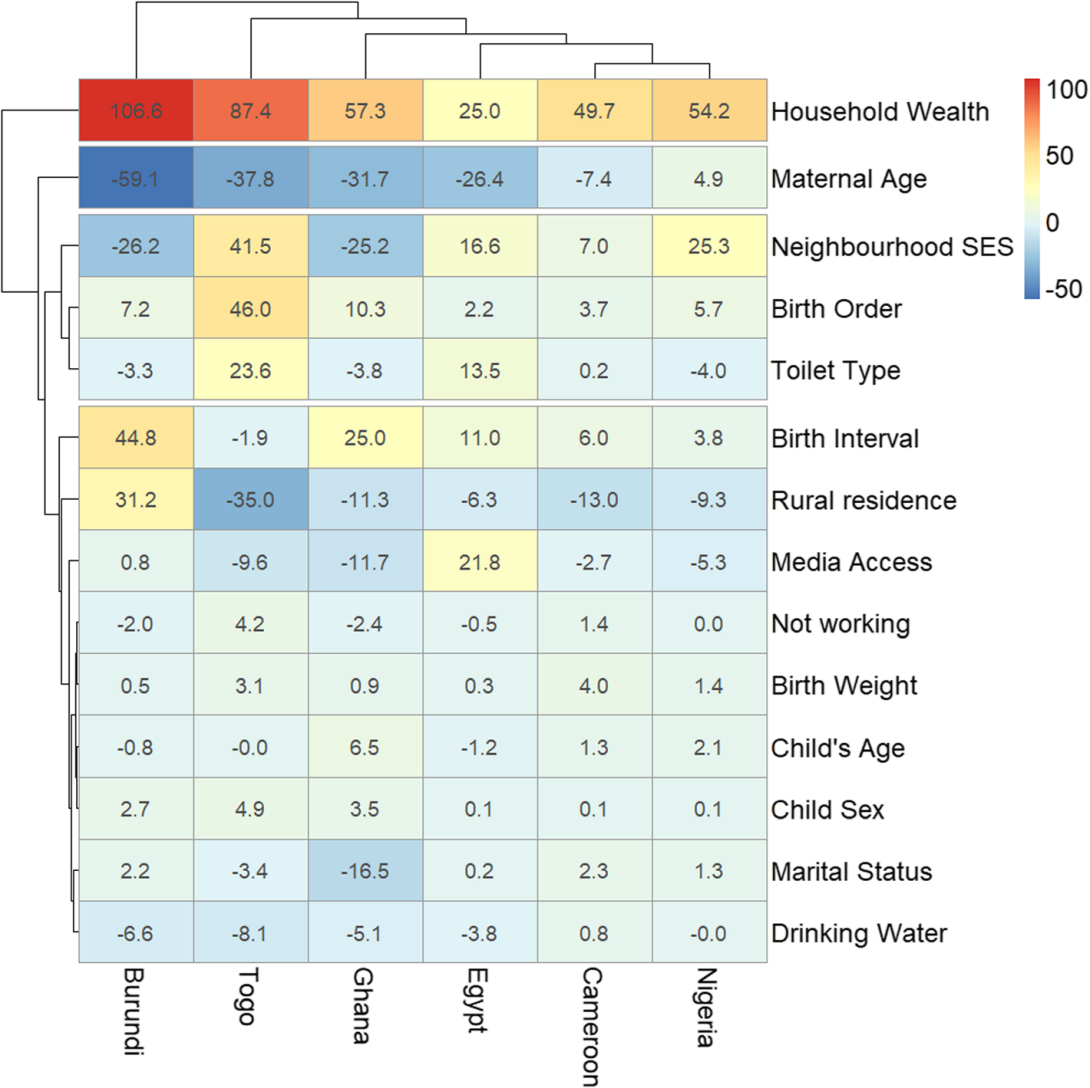
Contributions of differences in the distribution ‘compositional effect’ of the determinants of diarrhoea to the total gap between children from illiterate and literate mothers by countries

Descriptive and inferential statistics comprising of bivariable analysis and binary logistic multivariable Fairlie decomposition techniques were used for this study. The Z-test for equality of prevalence of diarrhoea among the children of illiterate and literate mothers within each country and region was conducted and reported in Table 1 while chi-square test of association between the explanatory variables and the outcome variable among the two groups of children were reported in Table 2. The risk difference (RD) in having diarrhoea was measured between U5C from illiterate mothers and those from literate mothers. An RD > 0 suggests that diarrhoea is more prevalent among children born to illiterate mothers (pro-illiterate inequality). Whereas, a negative RD (< 0) indicates that diarrhoea is prevalent among children born to literate mothers (pro-literate inequality). A meta-analysis of the prevalence of diarrhoea among both groups of children in each of the countries was carried out. We estimated the fixed effects as the weighted country-specific RD and the random effect as the overall RD irrespective of a child’s country (Fig. 1). Charts were used to show the distributions of the RDs (Figs. 2 and 3). Test of heterogeneity to ascertain that the 57 countries were different with regards to the odds ratio of having diarrhoea among children from illiterate and literate mothers was carried out, and a test of homogeneity of ORs among the 6 countries (with a significant odds ratio of having diarrhoea) to determine if the odds of having diarrhoea in those countries are homogenous or not. The heterogeneity in meta-analysis, measured by I^2^, refers to the variation in study outcomes between locations or countries; where a low I^2^ is an indication of low variability among locations. Lastly, the adjusted binary logistic regression method was applied to the 6 pro-illiterate countries to carry out a Fairlie decomposition analysis (FDA) on factors associated with the inequality and results presented in Fig. 4.

**Table 1:** description of demographic and health surveys data by countries, educational and diarrhoea prevalence among under-five children in LMIC, 2010–2018

Country	Year of Survey	Number of Clusters	Number of Under 5 Children	Weighted (%) Illiterate	Weighted Diarrhoea Prevalence (%)		
					Overall	^a^Illiterate	^b^Literate
All		63,378	796,150	33.5	**14.2	*15.8	13.4
Eastern Africa		6298	102,886	26.5	16.7	*15.3	17.2
Burundi	2016	554	12,431	46.8	22.5	22.9	22.2
Comoros	2012	252	2949	46.6	17.0	16.9	17.0
Ethiopia	2016	643	9916	65.9	11.9	*11.3	13.1
Kenya	2014	1593	19,889	11.9	15.4	14.2	15.5
Malawi	2016	850	16,246	13.5	21.9	*18.3	22.5
Mozambique	2011	610	10,157	37.3	11.2	11.0	11.4
Rwanda	2014	492	7474	14.7	12.2	*14.0	11.9
Tanzania	2015	608	9445	21.3	12.1	*9.4	12.8
Uganda	2016	696	14,379	10.9	20.0	18.8	20.2
Middle Africa		3081	71,630	31.4	19.0	19.4	18.8
Angola	2016	625	13,463	29.3	15.7	*14.5	16.2
Cameroon	2011	578	10,326	27.5	21.7	*30.3	18.4
Chad	2015	624	16,710	65.9	22.3	*19.9	26.7
Congo	2012	384	8723	7.3	19.3	*13.3	19.8
Congo DR	2014	536	16,994	18.9	17.0	*15.3	17.4
Gabon	2012	334	5414	6.2	16.8	*11.2	17.2
Northern Africa		874	15,458	17.7	14.0	*16.4	13.5
Egypt	2014	874	15,458	17.7	14.0	*16.4	13.5
Southern Africa		2544	25,529	5.3	15.5	16.9	15.4
Lesotho	2014	396	2824	0.9	12.2	8.1	12.3
Namibia	2013	536	4449	6.2	19.1	*15.4	19.3
South Africa	2016	668	3241	1.4	11.0	11.6	11.0
Zambia	2018	545	9311	10.3	15.5	17.3	15.3
Zimbabwe	2015	399	5704	1.2	17.1	23.6	17.0
West Africa		6285	139,382	61.6	14.7	*15.2	13.9
Benin	2018	555	12,512	65.5	10.5	10.5	10.6
Burkina Faso	2010	573	13,621	83.8	14.9	*14.4	17.2
Cote d’Ivoire	2012	351	6876	64.0	18.5	*16.6	21.7
Gambia	2013	281	7633	59.4	17.8	*16.4	19.7
Ghana	2014	427	5539	27.4	11.9	*14.4	10.9
Guinea	2015	401	7213	76.6	14.6	14.7	14.3
Liberia	2013	322	6806	41.8	22.7	22.0	23.2
Mali	2018	345	9171	73.0	17.2	17.6	16.4
Niger	2012	476	11,437	85.7	14.4	*14.0	16.4
Nigeria	2018	1389	30,603	45.0	12.8	*16.3	10.0
Senegal	2017	400	11,253	61.4	18.0	*18.7	17.0
Sierra Leone	2013	435	10,254	69.4	11.5	11.5	11.5
Togo	2013	330	6464	40.5	15.2	*17.3	13.8
Central Asia		682	10,216	1.7	10.2	*18.6	10.0
Kyrgyz Rep	2012	316	4222	0.0	5.2	0.0	5.2
Tajikistan	2017	366	5994	2.7	13.3	*18.7	13.1
South-Eastern Asia		1850	17,168	6.4	9.0	*12.5	8.7
Cambodia	2014	609	6934	13.7	12.9	13.4	12.8
Philippines	2017	1241	10,234	1.1	6.1	4.1	6.1
Southern Asia		33,053	322,219	33.3	11.5	*14.5	10.1
Afghanistan	2015	956	30,520	83.2	29.1	29.0	29.9
Bangladesh	2014	600	7541	16.4	5.7	5.9	5.7
India	2016	28,321	247,181	29.5	9.2	*9.5	9.1
Indonesia	2017	1967	17,155	1.0	14.2	13.8	14.2
Maldives	2016	265	3048	1.3	4.2	0.0	4.3
Nepal	2016	383	4827	34.1	7.7	8.6	7.2
Pakistan	2018	561	11,947	48.5	19.2	*17.5	20.8
Western Asia		2048	27,441	32.7	21.8	*30.7	17.5
Armenia	2016	306	1709	5.6	3.8	6.2	3.7
Jordan	2017	962	10,454	1.6	9.7	*4.6	9.7
Yemen	2013	780	15,278	55.1	31.4	31.5	31.3
Central America		1996	22,524	12.4	18.7	17.7	18.8
Guatemala	2014	856	12,038	18.5	19.2	*17.1	19.7
Honduras	2011	1140	10,486	4.8	18.0	20.4	17.9
South America		1401	9408	3.1	12.3	8.9	12.4
Peru	2012	1401	9408	3.1	12.3	8.9	12.4
Southern Europe		651	2745	1.4	6.1	0.0	6.2
Albania	2018	651	2745	1.4	6.1	0.0	6.2
Caribbean		1860	21,129	18.3	15.0	*12.0	15.7
Dominican Rep	2013	516	3560	2.3	18.2	13.3	18.3
Haiti	2016	449	6082	19.9	21.4	19.5	21.9
Myanmar	2014	440	4575	17.8	10.5	10.7	10.4
Timor-Leste	2016	455	6912	25.0	10.8	*7.5	11.9
Oceania		755	8415	25.5	15.4	15.0	15.6
Papua New Guinea	2016	755	8415	25.5	15.4	15.0	15.6

**significant at 5% chi-square test *significant at 5% test of equality of proportions between a and b

**Table 2:** Summary of pooled sample characteristics of the studied children and prevalence of diarrhoea in 57 LMIC

Characteristics	n	Weighted %	Weighted % Illiterate	Weighted Diarrhoea Prevalence (%)		
				Overall	Illiterate	Literate
Age						
Infant	164,438	20.7	31.7	*17.4	*18.6	*16.9
12–59 months	631,712	79.4	34.0	13.4	15.1	12.5
Sex						
Female	389,173	48.9	33.8	*13.8	*15.3	*13.0
Male	406,977	51.1	33.3	14.6	16.2	13.9
Household Head						
Male	669,287	84.1	34.7	*14.2	*16.0	*13.2
Female	126,863	15.9	27.4	14.5	14.2	14.6
Maternal age						
15–24 years	234,550	29.5	25.2	*16.4	*17.2	*16.1
25–34 years	414,014	52.0	33.9	13.2	15.3	12.2
35–49 years	147,586	18.5	46.6	13.4	15.4	11.7
Wealth Index						
Poorest	202,853	25.5	54.2	*15.1	*15.1	*15.2
Poorer	178,258	22.4	39.5	14.8	15.5	14.3
Middle	158,228	19.9	30.6	14.2	15.9	13.5
Richer	139,713	17.6	22.7	13.9	16.9	13.0
Richest	117,098	14.7	12.4	12.5	18.0	11.8
Employment						
Employed	526,983	66.2	32.2	*13.3	*14.1	*12.9
Unemployed	269,167	33.8	35.2	16.0	18.8	14.5
Media access						
No	316,993	39.9	56.1	*15.2	*15.3	*15.1
Yes	478,517	60.2	19.6	14.2	16.5	12.9
Drinking water sources						
Unimproved sources	175,663	22.8	48.7	*16.9	*17.9	*16.0
Improved sources	595,332	77.2	30.2	13.6	14.9	13.0
Toilet type						
Unimproved sources	388,386	50.4	48.7	*15.4	15.7	*15.1
Improved source	382,305	49.6	19.8	13.1	16.0	12.4
Marital status						
Never married	23,560	3.0	10.6	*16.9	13.9	*17.2
Currently Married	739,740	92.9	34.7	14.0	15.8	13.1
Formerly married	32,850	4.1	23.8	17.1	16.0	17.4
Cooking Fuel						
Unclean/Biomass	581,710	77.0	42.2	*14.9	*15.4	*14.5
Clean Fuel	173,921	23.0	11.8	12.4	19.7	11.4
Housing materials						
Unimproved sources	676,227	89.5	36.9	*14.8	*16.0	*14.2
Improved source	79,157	10.5	15.8	10.0	12.0	9.6
Weight at birth						
Average+	643,472	84.0	32.6	*13.6	*14.9	*13.0
Small	90,809	11.9	36.9	17.2	19.8	15.7
Very small	31,924	4.2	44.7	20.1	21.1	19.3
Birth Interval						
1st Birth	223,779	28.2	18.6	*13.1	*14.5	*12.8
< 36 months	308,310	38.8	43.1	15.0	16.1	14.2
36+ months	262,278	33.0	35.7	14.3	15.8	13.4
Birth Order						
1st	223,777	28.1	18.6	*13.1	*14.5	*12.8
2nd	192,088	24.1	24.7	13.1	14.3	12.7
3rd	129,829	16.3	35.2	14.2	14.9	13.8
4+	250,456	31.5	50.4	16.2	17.0	15.2
Location						
Urban	239,222	30.1	17.7	*13.4	15.9	*12.9
Rural	556,928	70.0	41.0	14.6	15.7	13.8
Neighbourhood SES						
Highest	159,709	20.1	9.2	*9.8	*11.9	*9.6
2	158,969	20.0	21.8	14.9	15.9	14.6
3	160,077	20.1	38.5	15.8	16.7	15.2
4	159,153	20.0	45.3	16.7	17.8	15.8
Lowest	158,242	19.9	55.1	14.0	13.8	14.2
Total	796,150	100.0	33.5	14.2	15.8	13.4

*significant at 5% chi-square test

### Decomposition analysis

Multivariable decomposition was used to quantify the contributions of risk factors to the differences in the prediction of an outcome of interest between two distinct groups in multivariate models [25]. The outputs from such regression models of group differences are partitioned into two components attributable to; (i) compositional differences between the two groups (endowments or explained differences) and (ii) a second component which is attributable to differences in the effects of the characteristics (coefficients or unexplained differences) [25]. The Blinder-Oaxaca Decomposition Analysis (BODA) [26–28] for linear regression models is the most famous of the models but it is not so reliable for non-linear models such as binary logistic regression [25, 29]. Other methods include the multivariate decomposition [25] and the Fairlie methods [29–33]. The Fairlie decomposition method is an extension of the BODA purposively developed for non-linear regression models including the logit and probit models. It was first developed in 1999 [34], updated in 2007 [30] with more simplifications to address path dependence issues and the method of incorporating sample weights in the technique in 2017 [29]. The Fairlie method has been reported to have more reliable estimates of non-linear regression models especially for the logit and probit models [29, 30, 33, 34] and was used in this study.

The decomposition analysis was carried out by calculating the difference between the predicted probability for one group (say the group of children from illiterate mothers (Group I)) using the other group’s (say the group of children from literate mothers (Group L)) regression coefficients and the predicted probability for group I using its regression coefficients [29]. The Fairlie decomposition technique is operationalized to constrain the predicted probability between 0 and 1.

The standard BODA of the two groups in linear regression is the average value of the dependent variable, *Y*, can be expressed as:

1 $$ {\bar {\mathrm{Y}}}^{IL}={\overline{\mathrm{Y}}}^I-{\overline{\mathrm{Y}}}^L=\overset{1^{st}}{\overbrace{\left[\left({\overline{\mathrm{X}}}^I-{\overline{\mathrm{X}}}^L\right){\hat{\beta}}^I\right]}}+\overset{2^{nd}\ }{\overbrace{\left[{\overline{\mathrm{X}}}^L\left({\hat{\beta}}^I-{\hat{\beta}}^L\right)\ \right]}} $$

Where ${\overline{\mathrm{Y}}}^J$ is the average probability of the binary outcome variable with a particular group *J*. The ${\overline{\mathrm{X}}}^J$ is a row vector of the average values of the explanatory variables and ${\hat{\beta}}^J$ is a vector of coefficient estimates for a particular group *J*. The numerical details have been reported [27, 35]. Fairlie et al. showed that the alternative decomposition in eq. (1) for a nonlinear equation *Y* = *F*(*X*), where *F* is the logistic cumulative distribution function, can be expressed as:

2 $$ {\overline{\mathrm{Y}}}^I-{\overline{\mathrm{Y}}}^L=\overset{1^{st}}{\overbrace{\left[\sum \limits_{i=1}^{N^I}\frac{F\left({X}_i^I{\hat{\beta}}^I\right)}{N^L}-\sum \limits_{i=1}^{N^L}\frac{F\left({X}_i^L{\hat{\beta}}^I\right)}{N^L}\right]}}+\overset{2^{nd}\ }{\overbrace{\left[\sum \limits_{i=1}^{N^L}\frac{F\left({X}_i^L{\hat{\beta}}^I\right)}{N^L}-\sum \limits_{i=1}^{N^L}\frac{F\left({X}_i^L{\hat{\beta}}^L\right)}{N^L}\right]}} $$

Where *N*^*J*^ is the sample size for group *J* [34]. Unlike in BODA where *F*(*X*_*i*_*β*) = *X*_*i*_*β*. $\overline{\mathrm{Y}}$ In eq. (2) is not necessarily the same as $F\left(\overline{\mathrm{X}}\ \hat{\beta}\right)$. In eqs. (1, 2), the 1st term is the part of the gap in the binary outcome variable that is due to group differences in distributions of *X*, and the 2nd term is the part due to differences in the group processes determining levels of *Y* . The 2nd term also captures the portion of the binary outcome variable gap due to group differences in unmeasurable or unobserved endowments. The compliment of eq. (2) is also valid.

The estimation of the total contribution is the difference between the average values of the predicted probabilities. Using coefficient estimates from a logit regression model for a pooled sample, ${\hat{\beta}}^{\ast }$, the independent contribution of *X*_1_ and *X*_2_ to the group’s gap is expressed as

3 $$ \frac{1}{N^L}X\sum \limits_{i=1}^{N^L}F\left({\hat{\alpha}}^{\ast }+{X}_{1i}^L{\hat{\beta}}_1^{\ast }+{X}_{2i}^I{\hat{\beta}}_2^{\ast}\right)-F\left({\hat{\alpha}}^{\ast }+{X}_{1i}^L{\hat{\beta}}_1^{\ast }+{X}_{2i}^I{\hat{\beta}}_2^{\ast}\right) $$

and

4 $$ \frac{1}{N^L}X\sum \limits_{i=1}^{N^L}F\left({\hat{\alpha}}^{\ast }+{X}_{1i}^L{\hat{\beta}}_1^{\ast }+{X}_{2i}^I{\hat{\beta}}_2^{\ast}\right)-F\left({\hat{\alpha}}^{\ast }+{X}_{1i}^L{\hat{\beta}}_1^{\ast }+{X}_{2i}^L{\hat{\beta}}_2^{\ast}\right) $$

respectively. The contribution of each variable to the gap is thus equal to the change in the average predicted probability from replacing the group *L* distribution with the group *I* distribution of that variable while holding the distributions of the other variable(s) constant. To obtain an accurate decomposition estimate, Fairlie et al. recommended the replication of the decomposition from a minimum of 1000 subsamples and finding the mean values of estimates from each separate decomposition [29]. Further numerical details have been reported [29, 31, 33, 34, 36].

We invoked the “Fairlie” Ado file in STATA 16 (StataCorp, College Station, Texas, United States of America) to carry out the decomposition analysis using the generalized structure of the model. We specified random ordering of the variables, sample weights and 10,000 replications of the decomposition to obtain optimal results that fully reflect the study population. Model fit was assessed using the Wald chi-square statistics and the log-likelihood ratio test. The R statistical software was used to draw all the Figures. All statistical tests were performed at 5% significance level. The results of this study are presented in Tables and Figures. All our estimates were weighted.

## Results

The LMIC included in this study are listed in Table 1 with their respective year of survey, the number of clusters, number of children, and percentage of children whose mothers are illiterate, the weighted prevalence of diarrhoea in all and specifically among children from illiterate and literate mothers. The overall proportion of children whose mothers were illiterate was 34%, none in the Kyrgyz Republic, 1% each in Lesotho and Indonesia and highest in Niger (86%). The overall diarrhoea prevalence was 14.2% (significantly different across countries at *p* < 0.001), with 15.8 and 13.4% (*p* < 0.001) among children from illiterate and literate mothers respectively (Table 1 and Fig. 1). The prevalence of diarrhoea among children whose mothers are illiterate ranged from 0.0% each in Albania and Maldives to 31.5% in Yemen while it ranged from 3.7% in Armenia to 31.3% in Yemen among children whose mothers are literate.

The z-test of equality of prevalence of diarrhoea among children from illiterate and literate mothers was statistically significant in 26 countries: Ethiopia (*p* < 0.001), Malawi (*p* < 0.001), Rwanda (*p* < 0.001), Tanzania (*p* < 0.001), Angola (*p* < 0.001), Cameroon (*p* < 0.001), Chad (*p* < 0.001), Congo (*p* < 0.001), Congo DR (*p* < 0.001), Gabon (*p* < 0.001), Egypt (*p* < 0.001), Namibia (*p* < 0.001), Burkina Faso (*p* < 0.001), Cote d’Ivoire (*p* < 0.001), Gambia (*p* < 0.001), Ghana (*p* < 0.001), Niger (*p* < 0.001), Nigeria (*p* < 0.001), Senegal (*p* < 0.001), Togo (*p* < 0.001), Tajikistan (*p* < 0.001), India (*p* < 0.001), Pakistan (*p* < 0.001), Jordan (*p* < 0.001), Guatemala (*p* < 0.001), and Timor-Leste (*p* < 0.001).

About 80% of the children were aged 12–59 months, 52% of their mothers were aged 25–34 years and 30% aged 15–24 years while 70% are from rural areas (Table 2). We found statistical significance(*p* < 0.05) in the association between all the explanatory variable considered with the occurrence of diarrhoea among all the children and also by the literacy status of the mothers except toilet types, marital status and place of residence that were not found to be significantly associated with the occurrence of diarrhoea among children whose mothers are illiterate. The prevalence of diarrhoea was consistently higher among the infants compared with those aged 12–59 months irrespective of their mothers’ educational status: 19% vs 15% among children whose mothers are illiterate and 17% vs 13% among children whose mothers are literate.

### Magnitude and variations in illiterate-literate inequality in diarrhoea

The risk differences (RD) in having diarrhoea among children whose mothers are illiterate and those whose mothers are literate across the 57 countries as well as the meta-analysis of the RD are presented in Fig. 1. The RD was significantly higher among children whose mothers are illiterate than those from literate mothers in Cameroon, Nigeria, Togo, Ghana, Egypt, Senegal and India; higher among children whose mothers are literate in 18 countries (Angola, Congo, Congo DR, Ethiopia, Niger, Guatemala, Burkina Faso, Gambia, Pakistan, Tanzania, Malawi, Maldives, Timor-Leste, Jordan, Cote d’Ivoire, Gabon, Albania, and Chad) and was insignificantly different in the 31 countries. The RD for the Kyrgyz Republic could not be computed (NA) because all mothers were literate in the country (Figs. 1, 2 and 3).

Irrespective of regions, the fixed effects of pro-illiterate RD in diarrhoea was widest in Cameroon (118.44/1000) while the fixed effects of pro-literate RD in diarrhoea was widest in Albania (− 61.90/1000). The pro-illiterate RD in diarrhoea was significant only in some countries in Africa. In Middle Africa, the only pro-illiterate difference was in Cameroon and the largest pro-literate RD was in Chad (− 67.84/1000). In Middle Africa, the only pro-illiterate difference was in Cameroon and the largest pro-literate RD was in Chad (− 67.84/1000). In Western Africa, the largest pro-illiterate difference was in Nigeria (63.11/1000) and the largest pro-literate RD was in Cote d’Ivoire (− 50.93/1000). The overall RD, that’s the random effects, per 1000 children were − 7.41/1000 children (95% confidence interval (CI): − 22.94-12.01), evidence of insignificant random effects and overall pro-literate inequality. The greatest contribution (weight) to the random effect was found in Nigeria and India at 2.2% each while the least was in Zimbabwe and Lesotho at 0.5% each (Fig. 1). In Figs. 2 and 3, we used the colours red, orange and blue to indicate statistically significant pro-illiterate inequality, insignificant inequality and statistically significant pro-literate inequality respectively.

### Relationship between prevalence of diarrhoea and magnitude of inequality

The relationships between the prevalence of diarrhoea and the magnitude of educational inequality, a function of RD, across the 57 countries are presented in Fig. 3. We categorized the countries into 4 distinct categories based on their prevalence of diarrhoea and whether or not the RD were small or large: (i) High diarrhoea prevalence and high pro-illiterate inequality countries such as Cameroon, Togo, and Senegal (ii) High diarrhoea prevalence and high pro-literate inequality countries such as Gabon, Congo and Chad (iii) Low diarrhoea prevalence and high pro-illiterate inequality countries such as Nigeria, Ghana and Egypt (iv) Low diarrhoea prevalence and high pro-literate inequality countries such as Maldives, Albania, and Jordan.

### Decomposition of educational inequality in the prevalence of diarrhoea

We first computed Mantel-Haenszel pooled estimate of the odds ratio (OR) of having diarrhoea while controlling for country among all the children as 0.99 (95% CI, 0.98–1.00) and tested the null hypothesis: OR = 1, we estimated z = 1.09 and *p* = 0.274 and (ii) Test of heterogeneity *X*^*2*^ = 784.64, degree of freedom (d.f.) = 56, and *p* = 0.000, I-squared (variation in odds ratio (OR) attributable to heterogeneity) = 92.9%.

The heterogeneity (I^2^) was computed among countries from the same regions. In Eastern Africa, the I^2^ was 79, 98% in Middle Africa, 44% in Southern Africa, 96% in Western Africa. 84% in Central America, 52% in South-Eastern Asia, 81% in Southern Asia, 78% in Western Asia, 72% in the Caribbean. Only one country each were studied in the other regions, hence the I^2^ cannot be computed. Also, all mothers in the Kyrgyz Republic were educated, I^2^ was not computed for Central Asia as it had only Tajikistan.

Of the 57 countries, we found statistically significant pro-illiterate odds ratio in only 6 countries, 14 showed pro-literate inequality while other 37 countries had no statistically significant inequality. The countries with pro-illiterate inequalities are Burundi (OR = 1.11; 95% CI: 1.01–1.21; *p* = 0.022), Cameroon (OR = 1.84; 95% CI: 1.66–2.05; p < 0.001), Egypt (OR = 1.26; 95% CI: 1.12–1.43; p < 0.001), Ghana (OR = 1.24; 95% CI: 1.06–1.47; *p* = 0.009), Nigeria (OR = 1.80; 95% CI: 1.68–1.93; p < 0.001), and Togo (OR = 1.21; 95% CI: 1.06–1.38; *p* = 0.005). While significance was found in Burundi in the binary logistic estimates, such was not found in India and Senegal that had significant pro-illiterate RDs in Fig. 3.

We then computed Mantel-Haenszel pooled estimate of the odds ratio (OR) of having diarrhoea among the children in all these 6 countries while controlling for the countries. We estimated OR = 1.47 (95% CI: 1.41–1.52) and tested the homogeneity of the ORs: *X*^*2*^ = 113.64, d.f. = 6, and *p* = 0.000. All the tests were significant.

The Fairlie decomposition analysis was carried out on only the 6 LMIC with significant pro-illiterate inequalities. Figures 4 show the detailed Fairlie decomposition analysis of the part of the pro-illiterate caused by compositional effects of the risk factors associated with diarrhoea among U5C from the pro-illiterate countries. The “explained” (compositional component) and the “unexplained” (structural component) portions of the inequalities are shown in red and blue colours respectively in Fig. 4. The lighter the red colour, the lower the percentage contribution of the “explained” portion of the inequalities attributable to specified characteristics in the individual countries. On the other way, the lighter the blue colour, the lower the percentage contribution of the “unexplained” portion of the inequalities attributable to specified characteristics in the individual countries. We found wide variations in the factors associated with the pro-illiterate and pro-literate inequalities across the countries. The underlying logistic models used in the decomposition analysis for each of the countries fitted the data. The Wald chi-square statistics with corresponding degrees of freedom for each country were Burundi (*X*^2^(25) = 114.10, *p* < 0.001), Cameroon (*X*^2^(25) = 158.61, *p* < 0.001), Egypt (*X*^2^(25) = 265.85, *p* < 0.001), Ghana (*X*^2^(25) = 63.87, *p* < 0.001), Nigeria (*X*^2^(25) = 317.61, *p* < 0.001) and Togo (*X*^2^(25) = 83.73, *p* < 0.001). These statistics suggest that the coefficients were not simultaneously equal to zero. In addition, the log-likelihood ratio tests were significant.

Across the 6 countries, the largest contributions to gaps in having diarrhoea among the two groups of children are household wealth status, maternal age, neighbourhood SES, birth order, toilet type, birth interval and place of residence. Of these most important contributors, there was evidence of clustering among neighbourhood SES, birth order and toilet type, as well as between birth intervals and place of residence. These two clusters then clustered together at higher levels, first by the maternal age and then by the household wealth status (Fig. 4). Among the countries, the contributions of the risk factors to the pro-illiterate inequalities in having diarrhoea was highest in Burundi, then Togo, Ghana, Egypt, Cameroon and least in Nigeria as shown in Fig. 4.

As shown in Fig. 4, there are values greater than 100%. This is because decomposition analysis works by separating the contributions of the factors into explained and unexplained which are arithmetically denoted as positives and negatives respectively. It is, therefore, possible to have both > + 100% and > − 100% as such will net out. Specifically, the largest contributions to pro-illiterate inequality in the prevalence of diarrhoea in Burundi were household wealth status (as it contributed 107% of the difference in diarrhoea prevalence among groups of children from literate versus illiterate mothers), maternal age (59%), birth intervals (45%), and rural residence (31%). Household wealth status (87%), followed by birth order (46%), neighbourhood SES (42%), maternal age (38%,), and rural residence (31%) were the greatest contributors to the inequalities in Togo. In Ghana, the main contributors to the educational inequalities were household wealth status (57%), maternal age (32%), neighbourhood SES (25%), and birth intervals (25%). Household wealth status, maternal age and media access were most significant in Egypt, household wealth status stood out in Cameroon while household wealth status and neighbourhood gave the highest explanation of the inequality in Nigeria. Other factors such as mothers’ current employment status, birth weight, the age and sex of the child, as well as mothers’ marital status and sources of drinking water had the lowest contribution to educational-related inequalities in the prevalence of diarrhoea across these countries. The clustering of the factors in the heat map refers to more closely related contributors to the inequalities.

## Discussion

The study aimed to decompose the factors associated with educational inequalities in the development of diarrhoea among under-five children across 57 low- and middle-income countries. The study was conducted to gain a better understanding of the endowment or compositional differences and the coefficient or unexplained differences that are associated with educational inequalities in the development of diarrhoea in the different countries especially those countries with pro-illiterate inequalities. We found variability in maternal literacy levels across the countries with regards to diarrhoea among U5C, and this may be attributed to the country’s existing nutrition strategies, policies, and interventions, as well as socio-economic status variations.

The adjusted random-effects showed that there are no significant differences in the development of diarrhoea among the children of the educated and the uneducated mothers generally irrespective of their countries of residence. Nonetheless, our analysis indicates an unequal balance between children of literate and illiterate mothers vis-a-vis the prevalence of diarrhoea within the countries. This is suggestive of maternal educational inequality being associated with the development of diarrhoea among children in LMIC. Of the 57 countries, children of illiterate mothers from 6 countries had significantly higher odds of diarrhoea, while diarrhoea prevalence was higher among children whose mothers were literate in 18 countries. Among the countries with statistically significant pro-illiterate inequalities, the risk difference of developing diarrhoea was widest in Cameroon (118 per 1000 children) and Nigeria (63 per 1000 children). The risk of diarrhoea has been previously correlated to maternal education, which can ultimately result in poorer health outcomes among children [37, 38]. This calls for an urgency of public health policies that are nutrition-specific, as well as programmes that can inform the design of tailored interventions that address the need to educate and train mothers to be able to make informed decisions about the nutrition of their children. Maternal education is also important as it influences household income and material resources within the household [37].

Maternal knowledge, practices and attitudes during childhood diarrhoea can be enhanced by education. According to Caldwell et al., child health can be improved solely by education enhancing the utilization of contemporary health services, or education may result in a host of complimentary behaviours that link to childcare and therefore contribute to the improvement of child health [39]. Besides, educational inequalities can make mothers unaware of a child’s nutritional requirements during diarrhoea, as diarrhoea hampers micronutrient and calorie intake, and therefore preventing adequate nutritional support and a greater likeliness of mortality. Diarrhoeal diseases and bacterial infections may affect child nutrition, and therefore clean water and sanitation are vital to reduce these infections and diseases [13]. Therefore, mothers must be included in health promotion, which includes information campaigns, outreach programs that highlight health awareness and preservation, as well as focus on risks, as part of a comprehensive health framework focusing on child nutrition. This requires concerted efforts from the community and political leaders, as well as scientists and healthcare personnel [40]. Maternal education protects against infant diarrhoea in the more economically and socially advantaged communities but does not affect the more disadvantaged communities. Nonetheless, a major setback in women’s educational inequalities especially in LMIC is that girls from poor families are most likely to remain out of school or have the strong likelihood to drop out even if they have a chance to start [41].

Closely related to the role of education in child health is media access to mothers as documented in an earlier study [42]. Research has shown that media is effective in the transmission of knowledge and information and therefore, should be included in national educational programs [43]. Efficient health education and media access over time can result in increased hygiene practice improvement, access and quality of water and sanitation, improved hygienic food preparation and access to nutritious food, as prevention measures for diarrhoea control in children [44],

We found wide variations in the factors associated with the pro-illiterate inequalities in the development of diarrhoea across the countries. The decomposition analysis indicates that of the 57 countries, only six countries, namely Burundi, Togo, Ghana, Egypt, Cameroon and Nigeria, had a statistically significant pro-illiterate odds ratio. To address and obtain a decreased cutback in maternal educational inequalities in diarrhoea, there has to be a greater understanding of the structural and contextual environment that the child lives. Previous studies identified the variations of country, community and individual stage factors associated with child health [44–46]. The greatest contributions to gaps in having diarrhoea among the two groups of children are household wealth status, maternal age, neighbourhood SES, birth order, toilet type, birth interval and place of residence across the six countries. The significance of these variables is consistent with previous studies, whereby diarrhoea was likely to occur in children whose mothers had little or formal education [47–51]. This is even though toilet types, marital status and place of residence were not found to be significantly associated with the occurrence of diarrhoea among children whose mothers are illiterate in the bivariate analysis. This finding could be attributed to different factors.

We found that children in rural areas are at a disadvantage of educational inequality in having diarrhoea than those living in urban areas. This is consistent with the literature. A study in Pakistan indicated that mothers in urban areas had better access to sanitation and healthcare facilities, improved water sources and also greater knowledge on the control and prevention of diarrhoea [52]. Literature indicates that essentially educated mothers have better understanding and knowledge regarding appropriate child feeding practices, hygiene, early diarrhoea signs and symptoms, which are major determinants for the occurrence of diarrhoea in children. It is also more likely that education impacts behaviour change at the household level and so awareness about methods of diarrhoea prevention and transmission may be greater [8, 13].

Our findings on household wealth, as the greatest factor associated with educational inequalities in the development of diarrhoea, are consistent with the literature [12, 53]. These studies indicate that there are higher risks of diarrhoea diseases in poorer households, while a child from a well-off household is likely better access to healthcare services, and therefore have better health outcomes. Income inequality is a major driver of educational inequalities among girls and women [54]. UNICEF and UNESCO were unanimous that the world’s low-income countries remain home to poverty and large income inequalities which invariably lead their girls to marry early without reasonable education [18, 55].

The mapping of the relationship between educational inequality and diarrhoea prevalence conducted in this study revealed that there are different categories of country viz-a-viz their level of diarrhoea prevalence and educational inequality. The mapping helped to identify the countries that need to urgently revisit, develop and re-strategize diarrhoea prevention strategies with a focus on the prevailing level of educational inequality in different countries. We found that countries such as Cameroon, Togo, and Senegal had a high prevalence of diarrhoea and high pro-illiterate inequality, while countries such as Jordan, Maldives and Albania had a low prevalence of diarrhoea and high pro-educated inequality. These disparities can be attributed to household wealth status, neighbourhood SES, current child nutrition policies and programmes at the country-level, as well as economic and political stability in different countries.

There are definite learning curves from countries with a low prevalence of diarrhoea and low pro-educated inequalities for countries with high diarrhoea prevalence and high pro-illiteracy inequalities. This can include realignment of or new nutrition policies and programmes for child health. Also, there is clear evidence of significant pro-educated inequality in some countries, this may be suggestive of the mother not having sufficient health education. That is, despite education, mothers’ knowledge, attitude and practice of diarrhoea prevention may be poor or insufficient. Further research to fully understand the structural and compositional factors that drive pro-literate inequalities in the development of diarrhoea is a pointer that diarrhoea prevention strategies should not be limited to only the uneducated mothers.

There are possibilities of path dependence between educational attainment and some of the risk factors. For instance, women with better education are more likely to belong to households in the richest wealth quintiles and vice versa [56]. On the other way round, people from poor households have a low likelihood of better education or even any education in some cases. Thus some contributors to the gap, are both causes and consequences of the inequalities.

It is striking that the risk of diarrhoea was higher among the children of educated women than among the children of uneducated mothers in countries such as Albania, Jordan, Gabon, Congo, Chad etc. This might have been affected by the type of food consumed. There is a possibility of “food poison” through “fast foods” which are more common among educated women than among uneducated women [57, 58]. Health education and promotion on how mothers could avoid or at least minimise the incidence of diarrhoea in those countries are necessary.

Although the level of education does not seem to be associated with under-5 diarrhoea in 37 countries in this study, as the gaps were insignificantly different, this does not necessarily translate to a lower risk of diarrhoea in those countries. The finding is an indication that the educational level of mothers does not significantly influence the risk of diarrhoea among children in those countries. In particular, countries such as Comoros, Uganda, Liberia, Haiti, Afghanistan and Yemen have a worrisome prevalence of diarrhoea. Stakeholders in these countries and others should focus on reducing the incidence of diarrhoea and also enhance adequate management of diarrhoea cases.

## Conclusion

The study identified inequalities in the prevalence of diarrhoea in children with variations in mothers’ literacy levels. Education-related inequalities in diarrhoea prevalence were identified and were explained by different risk factors at the individual, household and community levels. In explaining the inequalities in the prevalence of diarrhoea, our main determinant variable in this study, mothers’ literacy, highlights the importance of education to all countries, especially the girl child, as they are potential future mothers and their educational status may influence their health and that of their children. Maternal education is a vital factor that provides information and also enhances the autonomy level of a woman who can empower herself to provide good care of her child [59]. Education has the power to provide knowledge, change one’s attitude, practices and behaviours so that good health can be attained [59]. This can result in reducing the prevalence and morbidity and mortality associated with diarrhoea. In countries with pro-uneducated inequalities, urgent interventions are important to address the public health issue of diarrhoea as the second leading cause of death in children under five years.

### Recommendations for public health action

While enhancing the education of women, due attention must be given to enhancing individuals’ capacity for better livelihood, reduced fertility, childbirth at younger ages, improved toilet types, sufficient birth intervals, and rural residence. The study findings are important and concerted efforts must be made to design public health programmes and policy on childhood diarrhoea disease. There should also be collaborative efforts between governments and key stakeholders to focus on communities with a high level of illiteracy and should include programmes and interventions on access to healthcare, basic amenities such as water and sanitation, as well as hygiene practice techniques to address diarrhoea risk factors. Universal access to school for all women is vital so that the identified gap in diarrhoea among children with uneducated and educated mothers is lessened.

### Strength and limitation

A major limitation of our study is that the outcome variable is based on mothers’ ability to accurately recall the occurrence of diarrhoea among their under-five children without any means of verification. Correct identification of what diarrhoea is could be a potential bias. However, the provision of an exclusive description of how diarrhoea should be identified to the data collectors by the DHS could have limited the effect of this bias. Also, the data collectors were given a similar regimen of training across the countries, this would have improved both intra- and inter-country comparison. The very low levels of illiteracy among mothers in some countries could have reduced the precision of our estimates in those countries. Besides, our study has a strength of wide coverage of LMIC (57) using the DHS data that is comparable across countries. We quantified the magnitude of the explained and unexplained factors associated with educational-inequalities in the development of diarrhoea. Besides, we have used the Fairlie decomposition techniques which is more appropriate for binary outcomes although it is not necessarily an alternative measure of causality. However, it provided robust evidence of educational-related inequalities after controlling for the exposure variables.

## Data Availability

The data supporting this article is available at http://dhsprogram.com.

## Abbreviations

BODA: Blinder-Oaxaca Decomposition Analysis
CI: Confidence Interval
DHS: Demographic and Health Survey
FDA: Fairlie Decomposition Analysis
IRB: Institutional Review Board
LMIC: Low- And Middle-Income Countries
PSU: Primary Sample Unit
RD: Risk Difference
SES: Socioeconomic Status
U5C: Under-Five Children
UNICEF: United Nations International Children’s Emergency Fund
WHO: World Health Organization
